# The major nectar protein of *Brassica rapa* is a non-specific lipid transfer protein, BrLTP2.1, with strong antifungal activity

**DOI:** 10.1093/jxb/ery319

**Published:** 2018-08-31

**Authors:** Anthony J Schmitt, Andrew E Sathoff, Catherine Holl, Brittany Bauer, Deborah A Samac, Clay J Carter

**Affiliations:** 1 Department of Plant & Microbial Biology, University of Minnesota, St Paul, MN, USA; 2 Department of Plant Pathology, University of Minnesota, St Paul, MN, USA; 3 USDA-ARS, Plant Science Research Unit, St Paul, MN, USA

**Keywords:** *Arabidopsis thaliana*, *Brassica rapa*, lipid transfer protein, LTP, nectar, nectaries, nectary

## Abstract

Nectar is one of the key rewards mediating plant–mutualist interactions. In addition to sugars, nectars often contain many other compounds with important biological functions, including proteins. This study was undertaken to assess the proteinaceous content of *Brassica rapa* nectar. SDS-PAGE analysis of raw *B. rapa* nectar revealed the presence of ~10 proteins, with a major band at ~10 kDa. This major band was found to contain a non-specific lipid transfer protein encoded by *B. rapa* locus Bra028980 and subsequently termed BrLTP2.1. Sequence analysis of BrLTP2.1 predicted the presence of a signal peptide required for secretion from the cell, eight cysteines, and a mature molecular mass of 7.3 kDa. Constitutively expressed BrLTP2.1–GFP in Arabidopsis displayed accumulation patterns consistent with secretion from nectary cells. BrLTP2.1 was also found to have relatively high sequence similarity to non-specific lipid-transfer proteins with known functions in plant defense, including Arabidopsis DIR1. Heterologously expressed and purified BrLTP2.1 was extremely heat stable and bound strongly to saturated free fatty acids, but not methyl jasmonate. Recombinant BrLTP2.1 also had direct antimicrobial activity against an extensive range of plant pathogens, being particularly effective against necrotrophic fungi. Taken together, these results suggest that BrLTP2.1 may function to prevent microbial growth in nectars.

## Teaser

*Brassica* nectar contains a lipid-transfer protein with strong lipid-binding and antimicrobial activities *in vitro*. This is the first functional evaluation of a lipid-transfer protein in floral nectar.

## Introduction

Floral nectar is one of the primary rewards plants offer to pollinators to enhance visitation. While simple sugars are the primary solutes found in nectars, approximately 10% of nectar dry weight is represented by many classes of non-sugar metabolites (Lüttge, 1977). Depending on the species, nectars may contain amino acids, organic acids, terpenes, alkaloids, flavonoids, glycosides, vitamins, phenolics, inorganic ions, free fatty acids, and proteins (Heil, 2011; Roy *et al.*, 2017). These non-sugar compounds have been shown to perform a wide variety of functions, from acting as a deterrent to nectar robbers (Baker, 1978), to promoting pollination by attracting pollinators (Raguso and Pichersky, 1999; Carter *et al.*, 2006).

The production of a nutrient-rich nectar may be a double-edged sword. While nectar does facilitate pollinator visitation, it can also serve as an excellent growth medium for microbes. Indeed, microbial infection of plants via the nectaries is known to occur in cotton, bean, squash, apple, pear, aucuba, banana, pineapple, hawthorn, and gourds (Trapp, 1936; Temkin-Gorodeiski and Chorin, 1971; Rohrbach, 1986; Elada, 1988; Wilson *et al.*, 1990; Jaber and Vidal, 2009; Sasu *et al.*, 2010). In one of the best-known examples, fireblight in apple and pear trees is caused by colonization of nectar by *Erwinia amylovora* and subsequent invasion of the floral vasculature through the nectary glands (Buban *et al.*, 2003; Farkas *et al.*, 2006). In addition to pathogens, some relatively benign bacteria and yeasts are also known to grow in some nectars, which can impact pollinator behavior (Kevan *et al.*, 1988; Brysch-Herzberg, 2004; Vannette and Fukami, 2016).

It appears that some plants proactively limit microbial growth in nectar, usually via the secretion of antimicrobial proteins and secondary metabolites (Heil, 2011; Roy *et al.*, 2017). For example, two proteins secreted into tobacco nectar, nectarins I and V, have superoxide dismutase and glucose oxidase activities, which both produce hydrogen peroxide of up to 4 mM levels in nectar (Carter and Thornburg, 2000, 2004*a*, *c*). This high accumulation of H_2_O_2_ was found to be antimicrobial to a wide range of plant pathogens (Carter *et al.*, 2007). Many other examples of nectarins also exist in the literature (Heil, 2011; Roy *et al.*, 2017).

Approximately 75% of all crop species benefit from animal-mediated pollination (Klein *et al.*, 2007) and US pollinator-dependent crops alone have been estimated to have an annual value of nearly $29 billion (Calderone, 2012). For example, *Brassica* spp. are major worldwide crops, with varieties including canola, broccoli, cauliflower, turnip, and Chinese cabbage (bok choy) (Musgrave, 2000). Each year, over 23 million ha of canola and related varieties (e.g. oilseed rape) are planted globally, with up to 0.8 million ha planted in the USA alone. These species are largely self-incompatible and dependent on honeybees, wild bees, and flies to achieve full fecundity (Rahman, 1940; Downey, 1964; Downey *et al.*, 1970). Poor pollinator visitation has been reported to reduce yields of *Brassica* and unrelated species by up to 50% (Louveaux and Verge, 1952). Since nectar composition can greatly impact the frequency of pollinator visitation (e.g. Silva and Dean, 2000), full knowledge of the chemical constituents of nectar may have broad implications, ranging from a better understanding of the co-evolution of plant–pollinator and plant–microbe interactions, to increasing yields in multiple crop species. As such, here is described the identification and characterization of a lipid-transfer family protein with antifungal activity secreted into the nectar of *B. rapa.*

## Materials and methods

### Plant materials, growth conditions, and nectar collection

Rapid-cycling *B. rapa* (CrGC 1–33), obtained from Wisconsin Fast Plants at the University of Wisconsin, was used for nectar collection for the identification of nectar proteins. Arabidopsis thaliana ecotype Columbia-0 was used for protein localization studies. Plants were grown in individual pots on a peat-based medium with vermiculite and perlite (Pro-Mix BX; Premier Horticulture, Rivière-du-Loup, Quebec, Canada). All plants were grown under a 16 h day/8 h night cycle, photosynthetic photon flux of 150 µmol m^−2^ s^−1^ and temperature of 22 °C. Nectar was collected from *B. rapa* flowers via microcapillary pipette as previously described (Bender *et al.*, 2012).

### Chemicals and reagents

Unless noted otherwise, all chemicals were obtained through Sigma-Aldrich Chemical Co. (St Louis, MO, USA) or Thermo Fisher Scientific (Waltham, MA, USA).

### Nectar protein identification

Twenty-five microliters of raw *B. rapa* nectar was electrophoresed via standard one-dimensional 12% SDS-PAGE and silver stained with a method compatible for identification via liquid chromatography–tandem mass spectrometry (LC-MS/MS), as previously described (Shevchenko *et al.*, 1996). The major protein band at ~10 kDa was excised, rinsed twice in 300 μl ddH_2_O, dehydrated in 300 μl of 100% acetonitrile for 10 min, and dried in a SpeedVac. The dried gel slice was submitted to the Center for Functional Proteomics at the University of Albany, NY, USA for identification via LC-MS/MS. Briefly, the gel piece was washed, reduced, alkylated, and in-gel tryptic digested. Proteolytic peptides were extracted from the gel. The peptide mixture was concentrated and reconstituted in 5% formic acid for LC-MS/MS analysis. An Ultimate HPLC (Dionex, USA) was used for peptide separation on a Magic C18 column (5 μm, 100 μm ID×150 mm, Michrom Bioresources, Auburn, CA, USA), with a gradient based on solvent A (5% acetonitrile, 0.1% formic acid, 0.01% trifluoroacetic acid) and solvent B (85% acetonitrile, 10% isopropanol, 5% H_2_O, 0.1% formic acid, 0.01% trifluoroacetic acid). The flow rate was held at 250 nl min^−1^ with a 100 minute linear gradient ranging from 10% to 100% solvent B. Parent and fragmented peptides were recorded via a QSTAR XL MS/MS (Applied Biosystems, USA).

An MS/MS peak list was created using an Analyst ‘script’, mascot.dll. The peak list files were used to query *B. rapa* gene sequences using MASCOT 2.51 from Matrix Science (London, UK) with the following parameters: peptide mass tolerance, 0.3 Da; MS/MS ion mass tolerance, 0.3 Da; allow up to 1 missed cleavages. Variable modifications included deamidation (N, Q), oxidation (M), and carbamidomethylation (C). This analysis identified the major *B. rapa* nectar protein as a lipid-transfer protein encoded by the locus Bra028980. We subsequently termed this protein ‘BrLTP2.1’.

### *In silico* characterization of BrLTP2.1

The translated sequence of BrLTP2.1 was analysed *in silico* via PSORT (http://psort1.hgc.jp/form.html) with default parameters for the presence of an N-terminal signal peptide. Structural prediction of BrLTP2.1 was conducted via iTasser (Yang *et al.*, 2015; https://zhanglab.ccmb.med.umich.edu/I-TASSER/) using the predicted mature sequence (amino acids 27–98) via default parameters, with predicted models subsequently processed and viewed with DeepView/Swiss-PDBViewer v4.1.0.

Phylogenetic analysis of BrLTP2.1 was conducted via BLASTP (Altschul *et al.*, 1990) to identify homologs of known or predicted function, as well as all paralogs encoded by the *B. rapa* genome (Supplementary Tables S1, S2 at *JXB* online). The relationship between these proteins was subsequently analysed in Geneious Pro 5.4.7 using the Geneious Tree Builder with the following tree alignment parameters: cost matrix: Blosum62; gap open penalty: 12; gap extension penalty: 3; alignment type: global alignment with free end gaps. Tree builder option parameters included: genetic distance model: Jukes-Cantor; tree build method: Neighbor-Joining.

### BrLTP2.1 localization in Arabidopsis

Full-length BrLTP2.1, including the predicted signal peptide, was PCR amplified (primers in Supplementary Table S3) out of *B. rapa* genomic DNA and cloned into the *Xba*I and *Asc*I sites of pMDC85 (Curtis and Grossniklaus, 2003), which placed it downstream of the constitutive 35S CaMV promoter and upstream and in-frame with green fluorescent protein (GFP) (35S::BrLTP2.1-GFP). This construct was transformed into Arabidopsis Col-0 via *Agrobacterium*-mediated infiltration (Clough and Bent, 1998) and selected on solid 0.5× Murashige and Skoog medium supplemented with hygromycin (50 μg ml^−1^). Ten independent Arabidopsis transformants with similar GFP accumulation patterns were obtained. Plants confirmed to carry the construct were observed with an Olympus BX53 compound fluorescence microscope mounted with a SPOT Insight 4 MP CCD color digital camera and configured for the detection of GFP fluorescence. Sample preparation simply consisted of sepal removal from flowers to expose the nectaries prior to imaging, or the detachment of rosette leaves with a razor blade.

### Protein expression and purification

The predicted mature BrLTP2.1 (amino acids 27–98, minus signal peptide) was PCR amplified from genomic DNA and cloned into the *Bam*HI and *Xho*I restriction sites of the protein expression vector pET21a(+) in frame with the N-terminal T7-tag and C-terminal His6 tag, to form pCH1, which was verified by Sanger sequencing at the University of Minnesota Genomics Center. Two additional constructs containing mutations in cysteine 69 (C69A and C69Y) were also synthesized and cloned into the *Bam*HI and *Xho*I sites of pET21a(+) by GenScript Biotech (Piscataway, NJ, USA). Each expression construct was transformed into SHuffle^®^ T7 Express lysY *Escherichia coli* (C3030; New England Biolabs, USA), which allows for expression of cytosolic proteins with disulfide bonds. *Escherichia coli* cultures for protein expression were grown at 30 °C until log phase was reached (OD_600_ of ~0.6) and induced for expression of T7-BrLTP2.1-His6 with 1 mM isopropyl β-D-1-thiogalactopyranoside (IPTG). Induced cells grew for another 4 h at 30 °C and were subsequently harvested by centrifugation for protein purification. T7-BrLTP2.1-His6 was purified from *E. coli* with the HisPur™ Cobalt Purification Kit, 1 ml (Thermo Scientific™), according to the manufacturer’s instructions. Combined elution fractions were further purified by applying them to a 30K MWCO Amicon Ultra-4 Centrifugal Filter (Merk Millipore, Cork, Ireland), with the flow through containing BrLTP2.1 being collected. The flow through from the 30K MWCO filter was applied to a 3K MWCO Amicon Ultra-4 Centrifugal Filter to concentrate BrLTP2.1, which was lastly desalted with ~10 volumes of 25 mM NaPO_4_, pH 7.4. The concentration of the final purified BrLTP2.1 protein was determined by absorbance at 280 nm using predicted molecular masses and extinction coefficients. Purity was assessed by 4–20% SDS-PAGE and staining with PageBlue™ Protein Staining Solution (Thermo Fisher Scientific).

### Lipid-binding assays

The ability and preference for recombinant BrLTP2.1 to bind lipids was assessed by fluorescence spectroscopy and a ‘protein lipid overlay assay’. The fluorescence assay was performed essentially as previously described (Buhot *et al.*, 2004). In initial experiments, BrLTP2.1 concentration was kept constant at 2.5 μM in binding measurement buffer (BMB; 175 mM glucose, 0.5 mM K_2_SO_4_, 0.5 mM CaCl_2_, and 5 mM MES, pH 7.0), with the lipophilic fluorophore 2-*p*-toluidinonaphthalene-6-sulfonate (TNS) being added at final concentrations ranging from 0 to 10 μM. Blanks consisted of TNS in BMB without protein added. TNS binding to BrLTP2.1 was assessed through excitation at 320 nm and recording the emission at 437 nm. To test lipid binding specificity, free fatty acids and methyl jasmonate were added to a mixture of equimolar 2.5 μM BrLTP2.1 and 2.5 μM TNS in BMB, with TNS displacement from BrLTP2.1 being observed by a reduction in fluorescence at 437 nm.

A blot-based analysis of BrLTP2.1 binding to lipids consisted of a ‘protein lipid overlay assay’ as previously described (Dowler *et al.*, 2002), with minor modifications. In this case, 1 µl aliquots of 500 μM lipids dissolved in a 2:1:0.8 solution of methanol:chloroform:water were spotted onto nitrocellulose and allowed to dry at room temperature for 1 h. The blot was first incubated in blocking buffer [50 mM Tris-HCl, pH 7.5, 150 mM NaCl, 0.1% Tween 20 (v/v), 2 mg ml^−1^ fatty acid-free BSA] for 1 h at room temperature. His-tagged BrLTP2.1 was then added to the membrane in blocking buffer at a final concentration of 10 nM and incubated overnight at 4 °C with gentle rocking. The membrane was then washed 10 times for 30 min in TBS-T [50 mM Tris–HCl, pH 7.5, 150 mM NaCl, 0.1% Tween 20 (v/v)] and then incubated for 1 h at room temperature in a 1:2000 dilution of anti-His rabbit polyclonal antibody (GenScript Biotech) in blocking buffer. The membrane was then again washed 10 times for 30 min in TBS-T. A 1:5000 dilution of alkaline phosphatase-conjugated goat anti-rabbit secondary antibody (Thermo Fisher Scientific) was incubated in blocking buffer with the membrane for 1 h and then washed 10 times for 30 min in TBS-T. After washing, BrLTP2.1 binding to lipids was detected by developing the membrane in substrate buffer (100 mM Tris-HCl, pH 9.5; 100 mM NaCl; 5 mM MgCl_2_) containing 150 μg ml^−1^ nitroblue tetrazolium chloride and 300 μg ml^−1^ 5-bromo-4-chloro-3-indoxyl phosphate, *p*-toluidine salt.

### *In vitro* antimicrobial assays

A microplate assay was used to monitor fungal growth inhibition adapted from the previously described assay (Broekaert *et al.*, 1990). All fungal pathogen strains were retrieved from the University of Minnesota Mycological Culture Collection and were originally collected in Minnesota from commercial production fields. Fungal samples from *Fusarium oxysporum*, *F. graminearum*, *Bipolaris oryzae*, *Trichoderma viride*, *Alternaria solani*, and *Colletotrichum trifolii* were grown on potato dextrose agar plates for 1 week. Harvesting the spores was done by flooding the plates with sterile water and rubbing with a sterile rubber policeman. The spore suspensions were filtered, and spore densities were determined microscopically using a hemocytometer. Clear, flat bottom microplates were used with each well containing half-strength potato dextrose broth, approximately 2000 spores, and concentrations of BrLTP2.1 peptide up to 300 μg ml^−1^ in a total volume of 100 μl. The microplates were shaken on an orbital shaker and spores were allowed to sediment for 30 min before absorbance was measured. The absorbance of the wells was measured at 595 nm on a Synergy H1 microplate reader (BioTek, Winooski, VT, USA). Further absorbance measurements were carried out after 24-h and 48-h incubation periods. Absorbance values were calculated by subtracting the initial measurement from the final measurement. From these values, the amount of BrLTP2.1 needed to inhibit growth of the pathogens strains by 50% (IC_50_) was calculated.

A spread-plate method was used to quantify antibacterial activity of the BrLTP2.1. Bacterial lawns of *Pseudomonas syringae* pv. *tomato* were grown on LB plates for 2 days. The plates were flooded with sterile water, and a bacterial cell suspension was made by rubbing the plate with a sterile rubber policeman. Cultures were diluted with sterile water to an OD_600_ value of 0.1. In microcentrifuge tubes, 200 μl of bacteria was incubated with shaking for 3 h with concentrations of BrLTP2.1 up to 300 μg ml^−1^. After the peptide treatment, the bacteria were serially diluted, and 100 μl was plated in triplicate onto nutrient broth yeast extract (NBY) plates. After 48 h of incubation, the bacterial colonies were counted. From these values, the amount of BrLTP2.1 needed to inhibit growth of bacterial strains by 50% (IC_50_) was calculated.

### BrLTP2.1 heat stability assays and immunodetection

To test the heat stability of BrLTP2.1, 1 ml of *E. coli* cell culture was harvested 4 h after induction of BrLTP2.1 expression with IPTG and directly boiled for 5, 10, 30, and 60 min. Boiled samples were incubated on ice for 15 min and centrifuged at 17000 *g* for 10 min to precipitate denatured protein and cell debris. Remaining soluble proteins were evaluated by 4–20% SDS-PAGE as described above. In some cases, the boiled supernatant was further purified via affinity chromatography as described above. Western blot analysis of boiled and purified BrLTP2.1 was assessed with polyclonal rabbit anti-His (GenScript Biotech A00174) and alkaline-phosphatase-conjugated goat anti-rabbit (Thermo Fisher Scientific 31346) antibodies as previously described above in the protein lipid overlay assay.

## Results

### The major *B. rapa* nectar protein is a lipid-transfer family protein

**Fig. 1.**
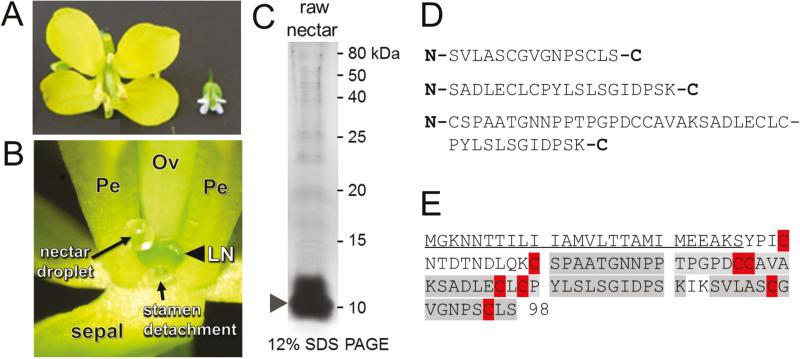
A lipid transfer protein (LTP) is the major protein in *Brassica rapa* nectar. (A) Whole *B. rapa* flower (left) beside one from its close relative, Arabidopsis. (B) Example of a nectar droplet collected from *B. rapa* flowers for protein identification. LN, lateral nectary; Ov, ovary; Pe, petal. A short stamen was removed from the flower to visualize the nectar droplet. (C) Protein profile of raw *B. rapa* nectar after separation by 12% SDS-PAGE and silver staining. The major protein band (arrowhead) was excised from the gel and processed for protein identification. (D) Peptides identified from the major protein band [arrowhead in (C)] by LC-MS/MS. (E) BLAST searches identified the major protein band as Bra028980, a putative lipid-transfer protein. Peptides identified by MS/MS are shaded in gray, cysteines are highlighted, and a putative signal peptide required for secretion from the cell is underlined.

*Brassica rapa* and Arabidopsis are close relatives that have similar floral and nectary structures (Fig. 1A; Davis *et al.*, 1998). As with Arabidopsis, the bulk of nectar in *B. rapa* is secreted by nectaries located at the base of short stamens (Fig. 1B; Davis *et al.*, 1998). Approximately 10 protein bands were observed in raw *B. rapa* nectar via SDS-PAGE, with a major band accumulating at ~10 kDa (Fig. 1C, arrowhead). LC-MS/MS analysis of the trypsinized gel slice containing this major band identified three peptides (Fig. 1D) that mapped to a predicted non-specific lipid-transfer protein (nsLTP) encoded by the *B. rapa* locus Bra028980.

Bra028980 encodes a full-length protein of 98 amino acids, which places it in the Type 2 class of nsLTPs. Following a nomenclature system recently outlined (Salminen *et al.*, 2016), we subsequently named the gene encoded by the Bra028980 locus as ‘BrLTP2.1’. The three peptides from the MS/MS analysis covered 57 of 98 amino acids (58%) of the predicted full-length coding region of BrLTP2.1 (shaded in gray in Fig. 1E). However, the online localization prediction tool PSORT predicted that BrLTP2.1 is secreted from cells via the presence of a 26 amino acid long N-terminal signal peptide. Thus, the peptides identified by LC-MS/MS covered 80% (57 out of 71 amino acids) of the mature, secreted protein (Fig. 1E). Like other nsLTPs, mature BrLTP2.1 contains eight cysteine residues (highlighted in red in Fig. 1E), which likely form disulfide bonds. Other physiochemical properties of BrLTP2.1 include a predicted mature molecular mass of 7.3 kDa and an acidic isoelectric point (pI) of 4.46.

### Heterologously expressed BrLTP2.1 is secreted from Arabidopsis cells

**Fig. 2.**
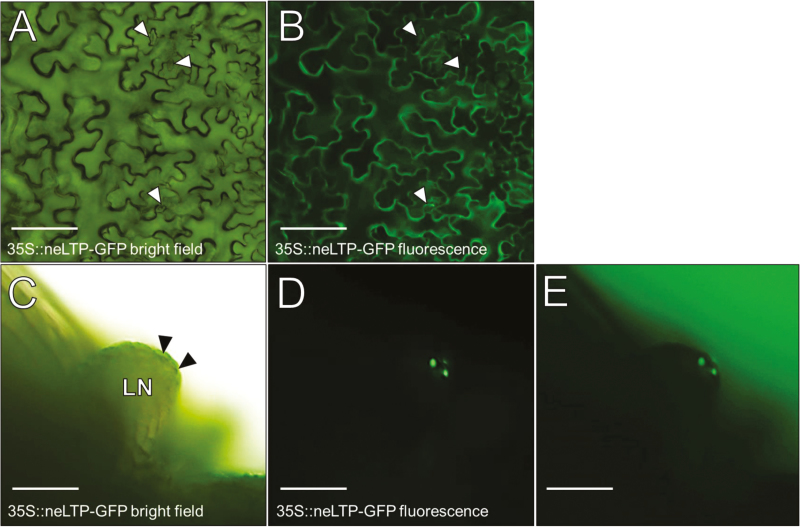
Constitutively expressed BrLTP2.1–GFP is secreted from Arabidopsis cells. (A, B) Full-length BrLTP2.1–GFP driven under control of the 35S-CaMV promoter leads to secretion from rosette leaf pavement cells. Arrowheads in (A) and (B) point to stomata. (C–E) BrLTP2.1–GFP preferentially accumulates in the stoma formed by guard cells in a lateral nectary (LN). Note that a similar accumulation of BrLTP2.1–GFP is not observed in the stomatal pores of rosette leaves (arrowheads).

To confirm that BrLTP2.1 is indeed secreted from cells, a 35S::BrLTP2.1-GFP construct was transformed into Arabidopsis. BrLTP2.1–GFP clearly outlined the pavement cells of rosette leaves (Fig. 2A, B), which is consistent with extracellular accumulation. Perhaps more strikingly, BrLTP2.1–GFP preferentially accumulated in the stomatal pores of lateral nectaries (Fig. 2C–E, arrowheads), which are the presumed locations of nectar secretion (Davis *et al.*, 1986, 1998). Note that the entire nectary fluoresces in 35S::BrLTP2.1-GFP lines and the images shown represent a relatively short exposure time when compared with the rosette leaf images (Fig. 2A, B), which is indicative of very high accumulation. Moreover, a similar pattern of GFP accumulation in stomatal pores was not observed in rosette leaves (arrowheads in Fig. 2A, B). The small size of the Arabidopsis nectary and associated nectar volume make it technically difficult to unambiguously determine if BrLTP2.1–GFP reaches the nectar itself. Therefore, while not conclusive, these results cumulatively suggest that heterologously expressed BrLTP2.1 is secreted into Arabidopsis nectar.

### Phylogenetic analysis of BrLTP2.1

**Fig. 3.**
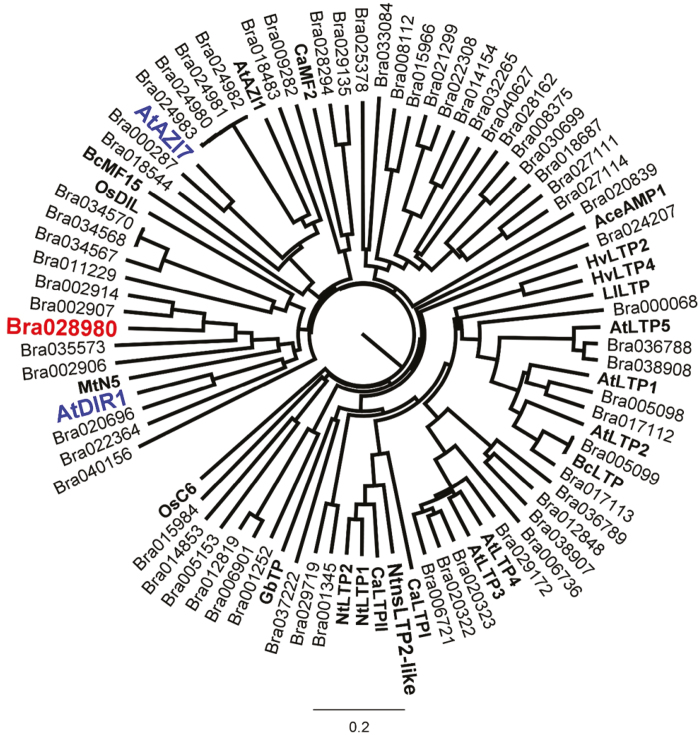
Phylogenetic analysis of Bra028980 (BrLTP2.1). The protein sequence of Bra02890 (BrLTP2.1) was subjected to CLUSTAL Omega multiple sequence alignment and tree analysis with all members of LTP family encoded by *B. rapa* genome, as well as select LTPs from other species with known or implicated biological function (in bold, see Supplementary Table S1 for detailed list). One of the nearest Arabidopsis LTPs with implicated function, AtAZI7, was also previously found to have enriched expression in nectaries by microarray analyses, suggesting conservation of BrLTP2.1 function, at least within the Brassicaceae.

In order to identify a potential biological function for BrLTP2.1, BLASTP and literature searches were used to identify close homologs, as well as nsLTPs with known or implicated functions (Supplementary Table S1). Included in this analysis were all 63 nsLTPs encoded by the *B. rapa* genome (Supplementary Table S2, contains sequences of all nsLTPs used for analysis). Of the nsLTPs with known functions, BrLTP2.1 was most closely related to Arabidopsis DIR1 (Fig. 3; 35% identity, 50% similarity), a protein involved in mediating systemic acquired resistance to pathogens (Maldonado *et al.*, 2002; Champigny *et al.*, 2013). In a previous report, both DIR1 and BrLTP2.1 fell into the same clade and were classified as ‘Type IV’ nsLTPs via a classification system based on sequence rather than functional similarity. Interestingly, BrLTP2.1 was also closely related to Arabidopsis AtAZI7, which we previously showed to have strong nectary-enriched expression profiles by microarray and RT-PCR analysis (Kram *et al.*, 2009). The biological function of AtAZI7 is currently unknown; however, its close paralog AtAZI1 was implicated in the long-distance priming of defense responses mediated by azelaic acid in Arabidopsis (Cecchini *et al.*, 2015). Azelaic acid, a saturated 7-carbon dicarboxylic acid, is produced in response to local infections and moves throughout the plant to promote systemic acquired resistance (SAR). Interestingly, azelaic acid production is dependent on the SAR regulator *ENHANCED DISEASE SUSCEPTIBILITY1* (*EDS1*) (Wittek *et al.*, 2014).

### *In silico* structural analysis of BrLTP2.1

**Fig. 4.**
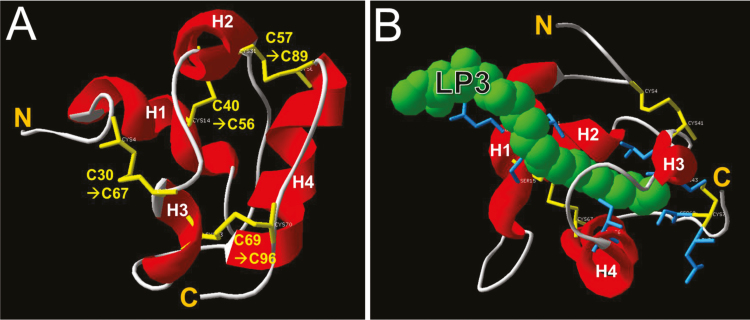
Structural prediction of BrLTP2.1. (A) AtDIR1, a close homolog to BrLTP2.1 involved in plant defense responses, was used as a template to model BrLTP2.1 structure. This analysis predicted the presence of four α-helices (labeled H1–H4 from N- to C-terminus) and four disulfide bonds. (B) Model of BrLTP2.1 with the lipid LP3 (1-stearoyl-*sn*-glycero-3-phosphocholine) bound. The specific sidechains predicted to coordinate lipid binding are Leu37, Gln38, Cys40, Ser15, Leu65, Leu68, Cys69, Ile82, Ser95, and Leu97. The models shown in both (A) and (B) were predicted by iTASSER using Arabidopsis DIR1 (2rknA) as a threading template. The sequence of the predicted mature BrLTP2.1 without signal peptide was used as the input, but the amino acid numbering includes the predicted 26 amino acid signal peptide.

The mature amino acid sequence of BrLTP2.1, without the signal peptide, was subjected to structural modeling at i-TASSER by using the crystal structure of DIR1 (2rknA; Lascombe *et al.*, 2008) as the threading template. Not surprisingly, and like most nsLTPs, BrLTP2.1 was predicted to have four α-helices and four disulfide bonds formed by eight cysteine residues (Fig. 4A). Moreover, a hydrophobic binding pocket complexed with a lipid ligand (1-stearoyl-*sn*-glycero-3-phosphocholine) was predicted based on the ligand-bound DIR1 crystal structure (Fig. 4B).

### BrLTP2.1 has lipid-binding activity

**Fig. 5.**
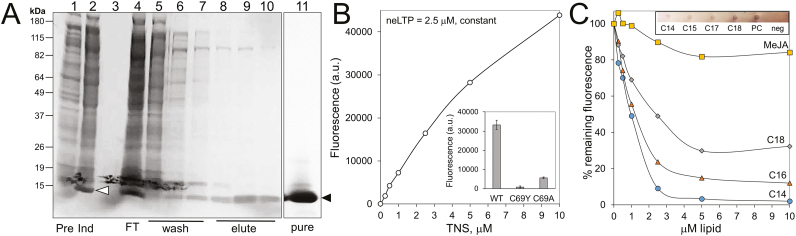
Heterologously expressed BrLTP2.1 has lipid binding activity. (A) Heterologously expressed and purified BrLTP2.1 from *E. coli*. Lane 1: pre-induction *E. coli* lysate; lane 2: 4 h post-induction lysate; lane 3: cell media; lane 4: flow-through from Co^2+^ affinity column; lanes 5–7: column washes; lanes 8–10: elutions with 300 mM imidazole; lane 11: pure protein after concentration and desalting. (B) TNS, a lipophilic fluorophore binds to BrLTP2.1. TNS concentration ranged from 0 to 10 μM, while BrLTP2.1 concentration was held constant at 2.5 μM, with excitation at 320 nm and emission recorded at 437 nm. Inset: TNS-dependent fluorescence in wild-type and two mutant versions of BrLTP2.1 (protein and TNS both at 2.5 μM). (C) Lipids present in *Brassica* nectars competitively displaced TNS from BrLTP2.1. TNS and BrLTP2.1 concentration were each held constant at 2.5 μM, while myristic acid (C14), palmitic acid (C16), stearic acid (C18), and methyl jasmonate (MeJA) ranged from 0 to 10 μM. Inset: Dot blot analysis of BrLTP2.1 binding to myristic (C14), pentadecanoic (C15), heptadecanoic (C17), and stearic (C18) acid, as well as phosphatidylcholine (PC). A 2:1:0.8 solution of methanol:chloroform:water, the solvent for all lipids, was used as a negative control (neg). BrLTP2.1 binding was detected with anti-His antibodies.

The structural analysis of BrLTP2.1, along with the fact that nsLTPs are known to bind a number of different lipids, led us to examine the binding activity of BrLTP2.1 *in vitro*. His-tagged BrLTP2.1 was heterologously expressed in *E. coli* and purified with a combination of affinity chromatography and size filtration (Fig. 5A). Importantly, we used a strain of *E. coli*, NEB SHuffle^®^ T7 Express lysY, which allows for the cytosolic formation of disulfide bonds, which are likely required for structural integrity. Therefore, we also purified two mutant versions of BrLTP2.1, both containing mutations in cysteine 69 (BrLTP2.1^C69A^ and BrLTP2.1^C69Y^) as negative controls.

We used the lipophilic fluorescent dye TNS to assess lipid binding to BrLTP2.1. TNS is weakly fluorescent in aqueous solution, but fluoresces brightly when bound to hydrophobic regions of proteins (Buhot *et al.*, 2004). In an initial test, BrLTP2.1 was held constant at 2.5 μM, with TNS added from 0 to 10 μM. Blanks containing TNS at the indicated concentrations in binding buffer (without BrLTP2.1) were subtracted from the observed fluorescence intensities from the test samples containing 2.5 μM BrLTP2.1. Total fluorescence was positively correlated to TNS concentration (Fig. 5B). Conversely, the two mutant versions, BrLTP2.1^C69A^ and BrLTP2.1^C69Y^, displayed minimal binding to TNS relative to the wild-type version of the protein (inset of Fig. 5B).

We previously identified free fatty acids in *Brassica* nectars accumulating at near millimolar levels, all of which were saturated (Bender *et al.*, 2013). To test if these lipids may bind BrLTP2.1, saturated free fatty acids with chain lengths of 14, 16, and 18 carbons (myristic, palmitic, and stearic acids) were added to solutions containing equimolar BrLTP2.1 and TNS (2.5 μM each). If BrLTP2.1 had a stronger binding affinity for a given lipid over TNS, then the observed fluorescence would decrease due to displacement of TNS via competitive binding. Each of the three free fatty acids tested strongly reduced TNS-dependent fluorescence (Fig. 5C), indicating that they displaced TNS from the BrLTP2.1 binding pocket. Moreover, BrLTP2.1 displayed a preference for myristic acid (C14) over the longer chain free fatty acids (based on the much larger decrease in fluorescence at lower concentrations). It is also important to note that maximal displacement of TNS from BrLTP2.1 occurred at 2.5 μM myristic acid, suggesting a 1:1 stoichiometry between myristic acid and BrLTP2.1 (which was held constant at 2.5 μM). Some LTPs also bind jasmonates (Bakan *et al.*, 2006), which are important in regulating nectary function (Radhika *et al.*, 2010; Stitz *et al.*, 2014; Wang *et al.*, 2014). Therefore, we tested the ability of methyl jasmonate (MeJA) to displace TNS from BrLTP2.1. MeJA did slightly reduce TNS-dependent fluorescence, though not nearly as much as the saturated free fatty acids tested. Lastly, a blot-based protein lipid overlay assay confirmed the binding of BrLTP2.1 to saturated free fatty acids, as well as phosphatidylcholine (inset of Fig. 5C).

**Fig. 6.**
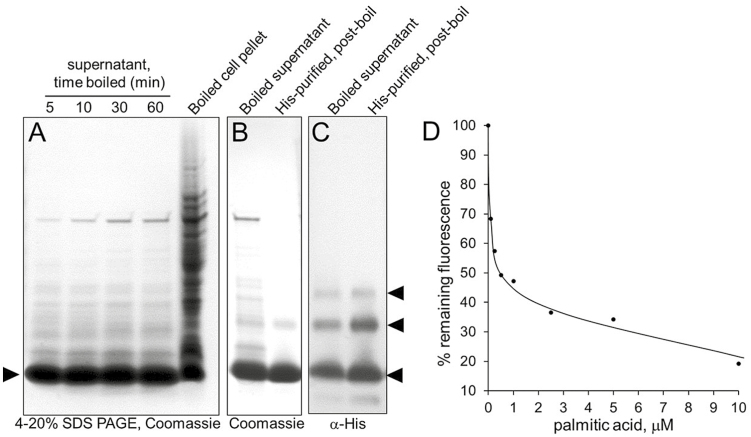
BrLTP2.1 is extremely heat stable. (A) One milliliter of *E. coli* cell culture was harvested 4 h after induction of BrLTP2.1 expression with IPTG and directly boiled for the indicated times. Boiled samples were incubated on ice for 15 min and centrifuged at 17000 *g* for 10 min to precipitate denatured protein and cell debris. Remaining proteins were evaluated by SDS-PAGE. (B) SDS-PAGE analysis of the clarified supernatant of cell cultures boiled for 15 min (left lane) and further purified BrLTP2.1 via Co^2+^ affinity chromatography (right lane). (C) Western blot analysis of boiled and purified BrLTP2.1 from (B) as detected by anti-His-tag antibodies. Arrowheads indicate the multiple bands corresponding to BrLTP2.1 post-boil and affinity purification. (D) Boiled BrLTP2.1 retains lipid-binding activity after purification [from (B)], as determined by displacement of TNS with palmitic acid.

Due to its small size and four disulfide bonds, BrLTP2.1 can be predicted to have significant heat stability, as has been shown for a number of other LTPs (Salminen *et al.*, 2016). Indeed, induced cultures of *E. coli* could be boiled for at least 60 min with no apparent loss or denaturation of BrLTP2.1 (Fig. 6A). The boiled protein was further purified via affinity chromatography (Fig. 6B) and analysed via Western blot (Fig. 6C). Interestingly, this analysis identified several isoforms of BrLTP2.1 (arrowheads in Fig. 6C), in spite of being boiled in Laemli loading buffer, which contains SDS and β-mercaptoethanol. This boiled and purified protein was also verified to still be able to bind free fatty acids, as determined by TNS displacement (Fig. 6D).

### BrLTP2.1 has broad antimicrobial activity *in vitro*

**Fig. 7.**
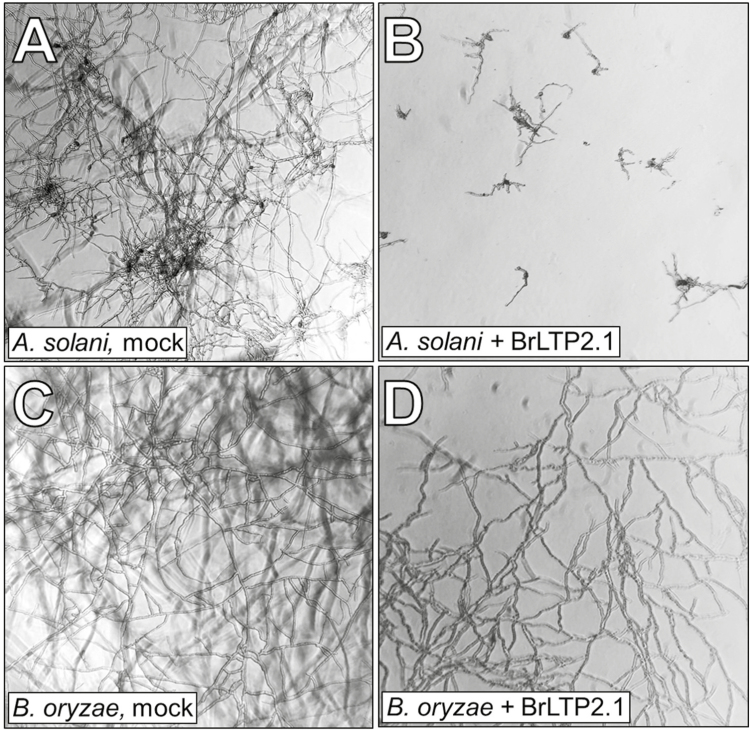
Heterologously expressed BrLTP2.1 has direct antimicrobial activity. Spores harvested from a battery of fungal plant pathogens were incubated with BrLTP2.1 from 0 to 300 μg ml^−1^ and monitored for growth over 48 h. In the examples shown, *Alternaria solani* (A, B) and *Bipolaris oryzae* (C, D) were either mock treated (A, C) or incubated with 50 μg ml^−1^ BrLTP2.1 (~5 μM; B, D). Summarized data are provided in Table 1.

A number of nsLTPs display antimicrobial activity *in vitro* (Salminen *et al.*, 2016). To test a potential role for BrLTP2.1 in limiting microbial growth, the recombinant protein was tested against a battery of fungal and bacterial plant pathogens. BrLTP2.1 displayed strong antimicrobial activity, particularly against necrotrophic fungi (Fig. 7; Table 1). The IC_50_ of BrLTP2.1 (the concentration at which microbe growth was reduced by half) was in the high nanomolar to low micromolar range against most fungal pathogens (Table 1). To verify that the observed antifungal activity was due to the purified BrLTP2.1, the mutant protein BrLTP2.1^C69Y^ was tested against two of these plant pathogens. Indeed, BrLTP2.1^C69Y^ displayed no activity against *Fusarium oxysporum* and ~30-fold lower activity against *Trichoderma viride* than the wild-type protein (Table 1). BrLTP2.1 also displayed activity against the single bacterial pathogen tested, *Pseudomonas syringae* pv. *tomato*, though the IC_50_ at ~35 μM was somewhat higher than for most fungal pathogens.

**Table 1.** BrLTP2.1 has direct antimicrobial activity against plant pathogens^*a*^

Microbe	Wild-type IC_50_		C69Y IC_50_	
	µg ml^−1^	µM	µg ml^−1^	µM
*Alternaria solani*	36.0	3.73	ND	ND
*Colletotrichum trifolii*	29.0	2.98	ND	ND
*Fusarium oxysporum*	140.2	14.4	No activity^*b*^	No activity^*b*^
*Fusarium graminearum*	248.3	25.5	ND	ND
*Trichoderma viride*	7.7	0.79	225.4	23.4
*Bipolaris oryzae*	16.7	1.72	ND	ND
*Pseudomonas syringae* pv. *tomato*	338.5	34.8	ND	ND

^*a*^ Fungal spores or diluted bacterial cultures were incubated with BrLTP2.1 (0–300 μg ml^−1^) and assessed for growth after 48 h.

^*b*^ Activity was tested at levels up to 300 μg ml^−1^.

ND, not determined.

## Discussion

Nectars often contain only a few major proteins (Roy *et al.*, 2017), therefore the finding of a single major protein, BrLTP2.1, in *Brassica* nectar (Fig. 1C) is not surprising. In a prior transcriptomic study we previously reported that BrLTP2.1 displays enriched expression in nectaries (Hampton *et al.*, 2010), which is consistent with the expression patterns of most nectarins (Carter *et al.*, 1999; Carter and Thornburg, 2003; Carter and Thornburg, 2004b,c; Seo *et al.*, 2013). Several nsLTPs from other species are also highly expressed in nectaries, including AZI7 in Arabidopsis (Kram *et al.*, 2009) and NaLTP1 and NaLTP2 in *Nicotiana attenuata* (Seo *et al.*, 2013). Based on sequence similarity alone, it does not appear that BrLTP2.1 is a direct ortholog of these other nectary-enriched nsLTPs, as other Arabidopsis nsLTPs share much closer sequence similarity to BrLTP2.1 at the amino acid level than does AZI7. Similarly, NaLTP1 and NaLTP2 do accumulate in *N. attenuata* nectar, but neither has been functionally characterized and they share only ~16% identity with BrLTP2.1. Therefore, it is difficult to extrapolate functional or biochemical conservation of nsLTPs with nectary-enriched expression.

Heterologously expressed and purified BrLTP2.1 has strong antimicrobial activity, particularly against fungal plant pathogens, with IC_50_ values in the high nanomolar to low micromolar range. This antifungal activity is on a par with other cysteine-rich peptides involved in defense, such as plant defensins (Lacerda *et al.*, 2014). These IC_50_ values, coupled with the apparently high concentration of BrLTP2.1 in nectar, strongly suggests that BrLTP2.1 is actively secreted into nectar as a broad-spectrum agent to limit fungal growth. Moreover, while the IC_50_ value for BrLTP2.1 against the bacterium *P. syringae* is on the high side, it is very rare for an nsLTP to have both antifungal and antibacterial activities – an onion nsLTP appears to be the only other known example (Cammue *et al.*, 1995). Finally, it is possible that an unidentified antimicrobial molecule from *E. coli* co-purified with BrLTP2.1, though several pieces of evidence strongly suggest otherwise: (i) the purified protein was extensively dialysed during the purification process; (ii) the fluorophore TNS readily binds at roughly equimolar concentrations to the same purified protein preparations used for the antimicrobial assays, suggesting the presence of an empty binding pocket; (iii) TNS itself is easily competitively displaced from BrLTP2.1 by free fatty acids at equimolar concentrations (Fig. 5), suggesting that TNS weakly binds BrLTP2.1 and that it is unlikely to displace other potential co-purifying molecules, if present; and (iv) related nsLTPs isolated from both natural and recombinant sources display similar antimicrobial activities (reviewed in Salminen *et al.*, 2016). These facts cumulatively indicate that it is highly unlikely that a small antimicrobial molecule from *E. coli* co-purifies with BrLTP2.1 and strongly suggest that the protein itself has direct activity. In a distinct but related point, the purified BrLTP2.1^C69Y^ mutant protein displayed greatly diminished antifungal activity relative to the wild-type protein (Table 1).

Since BrLTP2.1 is expressed in flowers prior to any apparent challenge by pathogens, this could indicate that this broad-spectrum antimicrobial activity contributes to non-host resistance. However, BrLTP2.1 also falls into a clade of nsLTPs that contains Arabidopsis DIR1 (Fig. 3; Li *et al.*, 2014). *dir1* plants exhibit no change in local responses to bacterial pathogens, but are defective in the development of systemic acquired resistance (SAR) to virulent strains (Maldonado *et al.*, 2002; Champigny *et al.*, 2013). AtAZI1 is another nsLTP that has been implicated in the azelaic acid-dependent development of SAR (Cecchini *et al.*, 2015). As mentioned above, AtAZI7, a close paralog to AZI1, is highly expressed in nectaries (Kram *et al.*, 2009). The relatively close relationship between BrLTP2.1, AtDIR1, and AtAZI7/AtAZI1 suggests an alternative role for BrLTP2.1 in defense signaling in *B. rapa*. Direct antimicrobial activity has not yet been reported for AtDIR1 or the AtAZI1 family of nsLTPs. Future studies will test if overexpression of BrLTP2.1 in plants leads to enhanced disease resistance.

The molecular mechanism through which nsLTPs inhibit microbial growth are currently unknown, but it is clear that nsLTPs can increase cell permeability, possibly through disruption of membrane structure (Sun *et al.*, 2008). A clue to the potential mechanism through which nsLTPs act may come from plant defensins. Defensins are small, cysteine-rich, antimicrobial proteins, like nsLTPs, that form large, multimeric pores in target membranes (De Coninck *et al.*, 2013). Intriguingly, nsLTPs are also known to form multimers *in vitro* (Pokoj *et al.*, 2010), and thus their antimicrobial activity may depend on a similar mechanism to that of some defensins.

An exhaustive study of potential ligands was not performed in our study, but BrLTP2.1 displayed an apparent preference for binding to shorter chain free fatty acids over longer ones, as well as over methyl jasmonate (Fig. 5C). These findings, along with the fact that the mutant BrLTP2.1^C69A^ and BrLTP2.1^C69Y^ did not bind TNS (Fig. 5C), suggests some degree of lipid ligand specificity by BrLTP2.1. Future studies will need to more thoroughly address the biochemical nature of BrLTP2.1 and its interactions with microbial membranes. While the physiological importance of the extreme heat stability of BrLTP2.1 (Fig. 6) is unknown, this characteristic could be very useful for purifying large amounts of the protein. It should also be noted that the extreme heat stability of BrLTP2.1 is not unique among nsLTPs (Salminen *et al.*, 2016), though a biological function for such stability has yet to be demonstrated.

Nectar is an inherently excellent growth medium for microbes, and therefore it is unsurprising that plants would secrete antimicrobial proteins, like BrLTP2.1, into nectar as a defense mechanism. For example, microbial infection of plants via the nectaries has been well documented in many species (Trapp, 1936; Temkin-Gorodeiski and Chorin, 1971; Rohrbach, 1986; Elada, 1988; Wilson *et al.*, 1990; Jaber and Vidal, 2009; Sasu *et al.*, 2010). Furthermore, yeast and bacterial communities inhabit nectars of a wide variety of plant species (Kevan *et al.*, 1988; Brysch-Herzberg, 2004; Vannette and Fukami, 2016). There is also growing evidence that these microbes can shape nectar composition in such a way that could deter pollinators through the consumption and metabolism of nectar compounds (Herrera *et al.*, 2008; Canto and Herrera, 2012; de Vega and Herrera, 2012; Vannette *et al.*, 2013; Good *et al.*, 2014). As such, nectar proteins are known to serve in a defensive capacity against nectar inhabiting microbes in both extrafloral (González-Teuber *et al.*, 2009, 2010; Heil, 2011) and floral nectars (Carter and Thornburg, 2004*a*; Roy *et al.*, 2017). In the constitutively secreted extrafloral nectar of two *Acacia* species, three pathogenesis-related enzymes were identified (chitinase, β-1,3-glucanase, and peroxidase), whose activities reduced fungal growth (González-Teuber *et al.*, 2010). Since BrLTP2.1 is the major protein secreted in *B. rapa* nectar (Fig. 1C) and has strong *in vitro* antifungal activity (Table 1; Fig. 7A–D), we suggest that the major role of this protein is to limit the growth of potentially deleterious microbial communities. This protective service may be necessary to maintain the integrity and quality of the nectar in order to effectively manipulate pollinators to achieve successful pollination. Future studies should aim to determine whether the presence or absence of this protein can indeed alter microbial growth in nectar and if this alteration in growth impacts nectar composition and pollinator preference.

Lastly, free fatty acids have been reported to accumulate to near millimolar levels in a few nectars (Kram *et al.*, 2008; Bender *et al.*, 2012), but how they are secreted is still unknown. Intriguingly, BrLTP2.1 appears to have strong affinity for the same saturated fatty acids (palmitic and myristic acids; Fig. 5C) that accumulate to high levels in *B. rapa* nectar (Bender *et al.*, 2012). Thus, a somewhat more speculative function of BrLTP2.1 is it may be involved in the transport of free fatty acids into nectars, which would not be dissimilar to the role that NtLTP1 plays in transporting lipids into glandular trichome secretions in tobacco (Choi *et al.*, 2012).

In summary, we have identified and partially characterized a *B. rapa* nectar protein, BrLTP2.1, with strong lipid-binding and antifungal activities *in vitro*. Future studies addressing its true biological role will depend on the evaluation of null mutant and overexpression plants. Such an analysis would determine if BrLTP2.1 limits microbial growth *in vivo* or if it is involved in the movement of lipids into nectar. Biochemical and biophysical approaches to probe the mechanism through which BrLTP2.1 binds lipids and prevents pathogen growth will also be beneficial to a general understanding nsLTP structure and function.

## Supplementary data

Supplementary data are available at *JXB* online.

Table S1. List of nsLTPs with known or putative biological functions used for phylogenetic analysis

Table S2. Sequences of nsLTPs used for tree building

Table S3. Oligonucleotides used in this study

Supplementary Tables S1-S3

## Abbreviations:

BrLTP2.1: *Brassica rapa* lipid-transfer protein 2.1
nsLTP: non-specific lipid-transfer protein
