# Supplementary material for: The major nectar protein of Brassica rapa is a non-specific lipid transfer protein, BrLTP2.1, with strong antifungal activity
Source: J Exp Bot. 2018 Aug 31;69(22):5587–97. doi: 10.1093/jxb/ery319 (PMC6255697; doi:10.1093/jxb/ery319)
Supplement: Supplementary Tables S1-S3 [file ery319_suppl_supplementary_tables_s1-s3.pdf]

Supplementary Table 1 -- List of nsLTPs with known or putative biological functions used for phylogenetic analysis

Gene	Accession #	Function
AtLTP1(At2g38540)	NP_181388.1	calmodulin-binding protein and cell differentiation
AtLTP2(At2g38530)	NP_181387.1	cuticle –cell wall interface integrity and etiolated hypocotyl permeability
AtLTP3(At5g59310)	NP_568905.1	freezing and drought stress
AtLTP4(At5g59310)	NP_568904.1	pathogen defense
AtLTP5(At3g51600)	NP_190728.1	pollen tube tip growth and fertilization
DIR1 (AT5G48485	NP_568699.1	system resistance signaling
AZI1 (AT4G12470.1)	NP_192984.1	systemic acquired resistance and induced systemic resistance, interacts with lipid derived azelaic acid
BraLTP1(Bra011229)	XP_009127438.1	epicuticular wax deposition, cell proliferation and flower development
nsLTP[Ginkgo biloba](DQ836633)	ABI26087.1	Proteinase inhibitor
LTP2(AAA03283)[barley, cv. Bomi]	Q43871.1	antibacterial
LTP4(Q43875.1) [barley, cv. Bomi]	Q43875.1	response to bacterial pathogens
BcMF15(ABQ63061.1 or EF600901)[Brassica rapa subsp. oleifera]	ABQ63061.1	microspore development
BcLTP(ABM69132.1 or EF216852)[Brassica rapa subsp. pekinensis]	XP_009143379.1	secrete and combine extracellular CaM
CaMF2(JF411954 or AEE92794.1)[Capsicum annuum]	AEE92794.1	pollen development
LTP(AF171094 or AAD46683.1)[Lilium longiflorum cv. Nellie White]	Q9SW93.1	pollen tube adhesion
Ace-AMP1 (AF004946.1 or AAB60896.1)[Allium cepa]	Q41258.1	antimicrobial
OsC6(AK064672 or BAG89152.1)[Oryza sativa Japonica Group]	XP_015615738.1	postmeiotic anther development
OsDIL(Os10g0148000) [Oryza sativa Japonica Group]	AAK91879.1	drought tolerance
MtN5(MTR_5g094210 or AET00633.1)[Medicago truncatula]	XP_003617674.1	efficient nodulation
NtLTP1 (AB625593 or BAK19150.1)[Nicotiana tabacum cv Xanthi]	XP_016433981.1	lipid secretion from glandular tissues
NtLTP2(Q03461.1) [Nicotiana tobacum cv Petit Havana SR1]	Q03461.1	Cell wall extension
NtnsLTP2-like	XP_009631888.1	antimicrobial resistance
CALTPI (AF2088232 or AAF23458.1) [Capsicum annuum]	AAF23458.1	Pathogen resistance and long distance signaling
CALTPII (AF208833 or AAF23459.1)[Capsicum annuum]	AAF23459.1	Pathogen resistance and long distance signaling

Supplementary Table 2 – Sequences of nsLTPs used for tree building

>Bra000068
MAGLMKLACFLVACMIVAGPITANAALTCASVVSNMARCISYLGGSETISGACCSGIRSINGLSR
TPSDRQIACGCLKRVATLPNINADRAAGLPNACGVSLPYNISKSANCTLYVDFNLSRYLSLSL

>Bra001345
MRSLFLLALFLVLAFHHGEAAVTCNNVVGDLYPCLSYVMQGGNSPSTNCCSGVRTLNSQAQT
TADRQSVCRCIKNAIGGASYSSSNLKNALSLPAKCGVNLPFSISPSTNCNR

>Bra005098
MAGLVKLACLVLACMIVAGPITSKAALSCGTVNTNVAACIGYLTQGGPLPRACCTGVSKLNSIAR
TTPDRKQACRCLKTAASALGSGLNAGRAAGLPKACGVNVPFPISTSINCNGHREISSVRMKLE
WIIPML

>Bra005099
MAGLMKLACLVLACMIVAGPITSNAALSCGTVSGYVAPCIGYLAQGAPALPRACCSGVTSLNNL
ARTTPDRQQACRCLVGAANAFPTLNAARAAGLPKACGVNIPYKISKTTNCNSVK

>Bra006721
MALALRFFTCLVLTVCIVASVDAAVSCGTVTSSLAPCANYLSKGGDVPPPCCAGVTKLNGMAQ
TTPDRQQACKCLQSAAKTVSGLDPSLAAGLPGKCGVSIPYPISMSTNCDNVK

>Bra006736
MASTLRFLTCLVTVCIVASVGAPISCGTVVQSMSPCITYLSGRMDLTAACCGGVRDLNAIAQTT
PDRQQTCKCLQAVAKKIPGFNQTRASDLPGKCRVSFPFPISISTNCDNVHHEDEEYMVYVQ

>Bra012848
MMFPSKITTCLLVLAVYMASPSESTITCGTVTSTLARCIGYLTNSGSLPSDCCVGVKSLNQMAQ
TTPDRRQVCECLKSAAKDITGLNTDLVATLPTTCGVSVPYPIRFSTNCDTISTAV

>Bra017112
MAGVMKLVCLVLACMIVAGPITANAALTCGTVNSNVAPCIGYITQGGPLPGACCTGVSKLNSMA
RTTPDRQQACRCLKTAASALGPSLNAGRAAGIPKACGVSVPFPISTNTNCNSVK

>Bra017113
MAGLMKLACLVLACMIVAGPITTNAALSCGTVSGNLAACIGYLTQNGPLPRGCCTGVTNLNNM
ARTTPDRQQACRCLVGAANSFPTLNAARAAGLPKACGVNIPYKISKSTNCNSVR

>Bra020322
MAIALRFFTCLVLTVCIVASVDAAITCGTVTSSLAPCATYLSSGGEVPPPCCAGVKKLNGMAQTT
ADRQQACKCLKAAAQGINPSLASSLPGKCSVSIPYPISMSTNCDNVK

>Bra020323
MTMGLKFFTCLVLTVCIAASVDAALTCGTVTSSLAPCATYLSKGGAVVPGPCCAGVKKLNDMA
QTTPDRQQACKCLKAAAKSINPSLASGLPGKCSVSIPYPISMSTNCDK

>Bra024983
MAYSKIALLLILNVIFFTLVSSNPVPYRKPTCKNALKFKVCANVLDLVKVSLPTRSKCCGLIKGLV
DLEAAVCLEAAVCLCTALKADLLGLKLNVPISLSVILNHCGKKVPSGFKCA

>Bra029172
MASALSFFTCLVLTVCIVASVDAAISCGTVTSNLVPCAGYLMKGGPVPASCCAGVSKLNSMAKT
TPDRQQACKCLKTAAKSVNPSLASSLPGKCGVSIYPISMSTNCNT

>Bra029719
MRSLLLALFLVLAFHRGEAAVSCNAVVGDLYPCLSYVVQGGNVPANCCNGIRTLNSQAQTPVD
RQGVCRCIKNAIGGVSFSSNNVNNAQSLPAKCGVNLPYSISPSTNCDR

>Bra036788
MEGLLKLSTLVIVCMLVSAPMASEAAISCGAVASNLGQCINYLTRGGFVPRGCCSGVRRLNSM
ARTTRDRQQACRCIQGAARALGSRLNPGRAARLPGACRVRIAYPISARTNCNK

>Bra036789
MAFASKIITCLLVLKVYMAAPAESHITCGIVTSTLAQCMGYLTNFFPVPSDYCCAEVKGLNQMAQ
TTPDRRQVCKCLKAVAKENKGFISIELVGTLPTICGVSVPYPFNFSTNCDTISTAV

>Bra037222
MSILKSLVTIFVLGIFLTPRYSESAISCSVVLSDLQPCVSYLTSGSGQPPETCCDGVRSLDAATTT
SADKKAACQCIKSVANSVTVKPELAKALASNCNASLPVDASSTVDCNTVG

>Bra038907
MTFASKIITCLLVLTIYIAAPTESHITCGTVTSTMTQCISYLTNGGPLPSSCCVAVKSLNQMAQTT
PDRRQVCECLKSAGKEIKGLNIDLVAALPTTCGVSLSYPIGFNTNCDSISIAV

>Bra038908
MEGFIKLSTLLIVCMLVSAPMAEAAISCGAVASNLGQCINYLTRGGFVPRGCCSGVQRLHSMAR
TTRDRQQACRCIQGAARALGSRLNPGRAARLPGACRVRISYPISARTNCNK

>Bra008112
MKFSCSKPVLFTCAILLLLIVAQENRVVAGQSCDPMQLIPCEEAILKGSKPSDTCCTRLNQQQH
CVCQYMKNPNFKSFLDSPNAKKIATDCHCPKPKC

>Bra008375
MKFTGVVCIAFVIVLVSASAPTKEVLEEKVACNSTEHITCIPALQSGSQPSAECCGKLKEEESCL
CGYIQNPLFSQYVTSANVHMVLVTCGIPYPSC

>Bra014154
MVKVMWGSSLALAAALLLVTVANIPVAEGVTCSPTELTSCSSAFMSASPPSATCCAKLREQKP
CLCGYLRNPALSQYVNSPNAKKVASSCNVATPKC

>Bra015966
MKASCTKPVLITCTILLLLIVAQENRVAAAEQCNPMQLMPCEDAIMKGSTPSNECCTRLKQQQH
CICQYMKNPNFKSFLNSPNAKMVASHCQCKPKC

>Bra018687
MNFTGAICIAFVIVLVSSLALTNAAVEDEKVLACNPKELNPCSPAVKTGSKPSTECCAMLKKEEP
CLCGYINDPVYGQYIKSKNAHKAFSSCGIPPLSC

>Bra021299
MEMIKAKWVSIVALAAIFLVVILVPAAEAVTCSPMQLSPCASAITSSSQTSALCCAKLKEQKPCLC

GYMRNRSLRRFVSSPNARKVSNRCKLPIPRC

>Bra022308
MVMIKATWVSIFAIAAVLLVILAPAAEAVTCSPMQLSPCAQAITSSSPPSALCCAKLKEQKPCLC
GYMRNPSLRRFVSSPNARKVSNRCKLPIPRC

>Bra025378
MKLTAIGLVAMVTIVVQLSPTMACDVKDLSPCLLPIAVFPESPTAACCQTLRDQGPCLCVFINNS
WIWIGPTLTSPNGHKLFAACQVPFPSCGN

>Bra027111
MKFTGAICIALVIVLVSSLDLTSAAVEEEIKVACVVTELIPCLESSIIGVHPYPECCVTLKAQQSCL
CGYIQNPVYGGFFKNAHSVFTGCGVPYPTC

>Bra027114
MKFTGAICIALVIVLVSSLDLTSAAVEEEIKVACVQTELIPCFVAAFIGSQPSAECCEKLKEQQSCL
CGYISNPVFGQFYKNAQNVFKACGVPYPTC

>Bra028162
MKFTTLMVITLVIIAMSSPVPIRATSVESFGEVAQSCVVTELAPCLPAMTTAGDPTTECCDKLVE
QKPCLCGYIRNPAYSMYVTSPNGRKVLDFCKVPFPSC

>Bra030699
MRFTGVVCIAFVIVLVSALAPTKADLEEKVACIPTELMTCIPALQTGSQPSAECCGKLKEQESCL
CGYIQNPLFSQYVTSENAHKILATCGIPYPTC

>Bra032265
MVKVMWVFVLALVAALLLVTVEKIPVAEGVTCSVTELSPCLAAFMSSSQPSASCCAKLREQKP
CLCGYMRNPGLRQYVTSPNAKKVSNSCKVASPNC

>Bra033084
MKFTTLASIAFVVVVLFSSTAAPINSQLIQSNSPCTTIDITGCVPAILYGAPLSPECCRNLNVQQP
CYCDFIKNAGLKPYITSPQGHAALASCGIPYPTC

>Bra040627
MVLTLMVFVILLTLFPAPNEAADTNVEAACDPKQLQPCLAAITGGGQPSGDCCAKLKEQQPCLC
GFSKNPAFAQYISSPNSRKVLTACGIPYPSC

>Bra009282
MEFLKSFTTILFVMFLAMSALETVPMVRAQQCLDNLSNMQVCAPLVLPGAVNPAPNSNCCIALQ
ATNKDCICNALRAATTFTTTCNLPSLDCGITI

>Bra028294
MVSISSSSKSSTIMKVVVMVAVVLVATVVDGQSCNTHLSGLNVCGEFVVPGADTTNPSAECCN
ALEAVPSDCICNTFRIASRLPTRCNIPTLSCN

>Bra029135
MIMKVAALVALVLVATEVDGQSCNRHLSGLNVCGEFVVPGADKTNPSAECCSALEAVPSECLC
NTMRIASRLPTRCSIPTLSCS

>Bra002906

MDENNTRTIVAALVIVFVSLVLMEEPTSIPLCNINANTLEKCRPAVTGNNPPLPGDACCIVLQAAD
LECVCKFKSHIPILATKSHKVHDLLRKCGIKTIPPACQDKTKVS

>Bra002907
MGKNNTKILITALVMIVTASMMIEEAKSVRICNVSTKDLKKCRPAVTGNNPPPPTPQCCQLAKAA
NLECLCPFLSRSGIDPSKIKALGANCGITKNPSCLPW

>Bra002914
MGKNNTKILVTALVMVVTAAMMIEEATSIPICGVNTNDLKKCSPAVTGNNPPPPTPQCCKVAKA
ANLECLCPYFTRSGLDTAKIKALGTNCGITKKPSCLPW

>Bra020696
MASKKVGVMVMMMMIVVVMAIFAERSVAIDLCGMTQSELNECKPAVSKENPTNPSTLCCDYLK
HADISCLCGYKNSPLLGSFGIDPALAAGLPTKCDMPNAPTC

>Bra020839
MAYVNKVSAVAAILFFAVAVAPLLAEPQTPMFPKMDPVCASLMPNLLEKCFSTVRETPTDDCCS
DLKSATTTQVTCLCDNYIANPAVVNFTGPYSAGITTKCGVFDKYSCNGSSNGT

>Bra022364
MEPNTKLVVITLVLALTLTAATGEFCGMSVSDLYSCKPYVQSKNPVTSAIDPKGPCCTALSKAD
FQCLCKQKTKTNPFLSSIDLDLASKLPEKCGLSGATC

>Bra028980 (BraNeLTP)
MGKNNTTILIIAMVLTTAMIMEEAKSYPICNTDTNDLQKCSPAVTGNNPPAPGPDCCAVAKSADL
ECLCPYLSLSGIDPSKIKSVLASCGVGNPSCLSW

>Bra035573
MGKNNTNILTQSTVLAMVLTAAIMVKEVSSLTICKIDINDMQKCRPAVIGINPPPPVNECCVVVRS
ANLECFCGFKFYLPILGIDPSKVAALVAKCDVTTIPPSCQVSKVLASRSLKNGA

>Bra005153
MKWCKFISVALMSLLITLASVEAAGECGRMPIGQAAASLSPCLAATKNPRGKVPPVCCAKVGAL
IRTNPRCLCAVMLSPLAKKAGINPGVAIAIPKRCNIRNRPSGKRCGRYIVP

>Bra014853
MASFMKFLCVLGLFLLVGTVVDGAGECGRSTPDNEAMKLAPCVGAAQDANAAVPGGCCAQIK
RFSQNPKCLCAVLLSDTAKASGVQPEVALTIPKRCNFANRPVGYKCGVLRVQIHGLYDEALEFT
TTS

>Bra011229 (BraLTP1) (XP_009127438.1)
MATGSRVLIGLAMILIISGELLVPGQGTCQGDIEGLMRECAVYVQRPGPKVNPSAACCKVVKRS
DIPCACGRITPSVQKMIDMNKVVLVTSFCGRPLAHGTKCGSKSVIN

>Bra034567
MGSGMITVMVVAIAFFMIGSDNVNMATAQLCGANLSGLVNECQRYVSNAGPNSPPPSRSCCA
LIRPIDIPCGCRYVTRDVMNTFDMDKLIYVARSCGKKIPSGYKCGSYTIPAA

>Bra034568
MASGRIIIMVVAIAFFMIGSDNVNVATAQFCGANVSGLMNECQRYVSNAGPNSPPPSRSCCALI
RPIDVPCACRYVSRDVTNYIDMDKVVYVARSCGKKIPSGYKCGSYTIPAA

>Bra034570
MASGRIIIMVVAIAFFMIGSDNVNVATAQFCGANVSGLMNECQRYVSNAGPNSPPPSRSCCALI
RPIDVPCACRYVSRDVTNYIDMDKVVYVARSCGKKIPSGYKCGSYTIPAA

>Bra015984
MEICKFLTVIFVAIVVLYSVQAAEQGGDHHSMACMQKLMPCQNYIHAVNPAPPASCCGPMKEIV
EKDSKCLCTVFNNPELLKSLNLTKENALDLPKACGVNPDVSICTKTACKFFIIFSTKIPHIYFI

>Bra001252
MILMVLVESGLLKEATAHPCGRTFLSALIELVPCTLSVVPFSTLSPNEPCCTAIKTLGQPCLCVIA
NGPSIPGVDHTLALQLPGKCSANFPPCN

>Bra006901
MKAMRVGLAMALLMTITVLTIVTAQLEDQQPPPPMLPEEEVGGCSRTFFSALVQLIPCRAAVAP
FSPIPPTQSCCSAVVTLGRPCLCLLANGPPLSGIDRSMALQLPQRCSANFPPCDIIN

>Bra012819
MMMRPMRVGLAMALLMTITVLTIVIAQQEDQQPPPPMLPVEEVGMCSRTFFSALVQLIPCRAAV
APFSPIPPTESCCSAVVTLGRPCLCLLANGPPLSGIDRSMALQLPQRCFANFPPCDVIN

>Bra000287
MAAKTSTILITIFLVINLLFLNFIPLVVAENTCPRDQLKLSTCANILNLINLNLGAPAMRPCCSVLLG
LIDLDIALCFCSALKLSILGITTNTPIHLNLALNACGGTLPDGFRCPT

>Bra018483
MAPRTSLALFLFLNLLFFTYTTAQGTCPRNALQIGACTNVLNAIDLTLGNPPPPVPPCCSLIAGLA
DLEAAVCLCTALDVNVLGNNVHLPIDISVLLNACSRFAPPSFQCP

>Bra018544
MAISKAFPLLLVLLLVLNSTFSFCHAVKQCPPPRKQSSMKCPRDTVKFGVCGSWLGLVHEVIGT
PPSQECCSLVKGLADLEAALCLCTALKTSLLGVAPVKLPVALTLLLNSCGKTLPQGFVC

>Bra024980
MAYSKIALLLILNVIFFTLVSSNPVPYRKPTCKNALKFKVCANVLDLVKVSLPTRSKCCGLIKGLV
DLEAAVCLCTALKADLLGLKLNVPISLSVILNHCGKKVPSGFKCA

>Bra024981
MAYSKIALLLILNVIFFTLVSSNPVPYRKPTCKNALKFKVCANVLDLVKVSLPTRSKCCGLIKGLV
DLEAAVCLCTALKADLLGLKLNVPISLSVILNHCGKKVPSGFKCA

>Bra024982
MAYSKIALLLILNVIFFTLVSSNPVPYRKPTCKNALKFKVCANVLDLVKVSLPTRSKCCGLIKGLV
DLEAAVCLCTALKADLLGLKLNVPISLSVILNHCGKKVPSGFKCA

>Bra024207
MKIHRAIILVTLLALIKTAVSQLQSIEQCREVFDSFMPCMGFVEGIFEQPSPQCCRGVSHLNNVV
KFKTPGSRKNEQGTGQLERVCECIEMMGKSDHLPFLASAINNLPPLCSLSLSFPISVGMDCSQF
RNMKELDAEKVN

>Bra040156

MMMMRVAFAMTCMLFAITTADKGDRPWPRECLEVANVMVEECKLFFVEQESPPTAECCGWF
SSRREKAKDRRRICRCMEFLTTAFEAIKPSVLALSDQCHFGGGFPISKNHACACKLHSFDQTL

>AtLTP1 (NP_181388.1)
MAGVMKLACLLLACMIVAGPITSNAALSCGSVNSNLAACIGYVLQGGVIPPACCSGVKNLNSIAK
TTPDRQQACNCIQGAARALGSGLNAGRAAGIPKACGVNIPYKISTSTNCKTVR

>AtLTP2 (NP_181387.1)
MAGVMKLACMVLACMIVAGPITANALMSCGTVNGNLAGCIAYLTRGAPLTQGCCNGVTNLKNM
ASTTPDRQQACRCLQSAAKAVGPGLNTARAAGLPSACKVNIPYKISASTNCNTVR

>AtLTP3 (NP_568905.1)
MAFALRFFTCLVLTVCIVASVDAAISCGTVAGSLAPCATYLSKGGLVPPSCCAGVKTLNSMAKT
TPDRQQACRCIQSTAKSISGLNPSLASGLPGKCGVSIPYPISMSTNCNNIK

>AtLTP4 (NP_568904.1)
MAFALRFFTCFVLTVFIVASVDAAITCGTVASSLSPCLGYLSKGGVVPPPCCAGVKKLNGMAQT
TPDRQQACRCLQSAAKGVNPSLASGLPGKCGVSIPYPISTSTNCATIK

>AtLTP5 (NP_190728.1)
MEGLLKLSTLVIVCMLVTAPMASEAAISCGAVTGSLGQCYNYLTRGGFIPRGCCSGVQRLNSLA
RTTRDRQQACRCIQGAARALGSRLNAGRAARLPGACRVRISYPISARTNCNTVR

>AtDIR1 (NP_568699.1)
MASKKAAMVMMAMIVIMAMLVDTSVAIDLCGMSQDELNECKPAVSKENPTSPSQPCCTALQH
ADFACLCGYKNSPWLGSFGVDPELASALPKQCGLANAPTC

>AtAZI1 (NP_192984.1)
MASKNSASLALFFALNILFFTLTVATNCNCKPSPKPKPVPSPKPKPVQCPPPPRPSVPSPNPRP
VTPPRTPGSSGNSCPIDALKLGVCANVLSSLLNIQLGQPSSQQCCSLIQGLVDVDAAICLCTALR
ANVLGINLNVPISLSVLLNVCNRKLPSGFQCA

>AtAZI7 (NP_192990.1)
MSMAPKTSTTLALFLVTNILFLNLITLSCADNTCPRDVLKLSTCSNVLNLINLKLGAPAMRPCCSIL
FGLIDLDVAVCLCTALKLSLLGITIDTPIHLNLALNACGGTLPDGFRCPT

>GbTP (ABI26087.1)
MMKISWQLWLLVAFAVMVCVWTPLSTAAPGCDTVDTDLAPCISYLQTGTGNPTVQCCSGVKT
LAGTAQTTEDRKAICECIKTAAIRVKPVANAVKSLPGLCSVTLPFPISIATDCNKIV

>HvLTP2 (Q43871.1)
MARTAATKLALVALVAAMLLVAADAAITCGQVSSALGPCAAYAKGSGTSPSAGCCSGVKRLAG
LARSTADKQATCRCLKSVAGAYNAGRAAGIPSRCGVSVPYTISASVDCSKIH

>HvLTP4 (Q43875.1)
MARAAATQLVLVAMVAAMLIVATDAAISCGQVSSALSPCISYARGNGAKPPVACCSGVKRLAG
AAQSTADKQAACKCIKSAAGGLNAGKAAGIPSMCGVSVPYAISASVDCSKIR

>BcMF15 (ABQ63061.1)
MKLISLMCIAFVILLTSFPATAITFNPACIKNHDTCGPLVAVKGRRWRPECCKIWSGNVLPETRQ
CACYVLKHSLFGNGVLPLILAKCKLGGIEQFKCSEVET

>BcLTP (XP_009143379.1)
MAGLMKLACLVLACMIVAGPITSNAALSCGTVSGYVAPCIGYLAQGAPALPRACCSGVTSLNNL
ARTTPDRQQACRCLVGAANAFPTLNAARAAGLPKACGVNIPYKISKTTNCNSVK

>CaMF2 (AEE92794.1)
MADVKSSVVSLFLLGLLVVVLQSGVIECQPQICNPSLTSLNVCAPFVVPGAPSASAECCTALQSI
NHGCMCDTMRIAAQIPAQCNLPPLSCAAN

>LlLTP (Q9SW93.1)
MARSSAVCFLLLLAFLIGTASAITCGQVDSDLTSCLGYARKGGVIPPGCCAGVRTLNNLAKTTPD
RQTACNCLKSLVNPSLGLNAAIVAGIPGKCGVNIPYPIRMQTDCNKVR

>AceAMP1 (Q41258.1)
MVRVVSLLAASTFILLIMIISSPYANSQNICPRVNRIVTPCVAYGLGRAPIAPCCRALNDLRFVNTR
NLRRAACRCLVGVVNRNPGLRRNPRFQNIPRDCRNTFVRPFWWRPRIQCGRINLTDKLIYLDA
EE

>OsC6 (XP_015615738.1)
MAPSKSTAAAGVLLVLLVAAAGGGAEAAATTCVASLLELSPCLPFFKDKAATAAPEGCCAGLSS
IVKGEAVCLCHIVNHTLERAIGVDIPVDRAFALLRDVCRLSPPADIISTCANEKGGVPPLYSCPAP
SA

>OsDIL (AAK91879.1)
MSQLKSSSLAALLIFLLAVFTTAAAAAGTECQNDVEVLKTTCYKFVEKDGPKLQPSPDCCTSMK
GVNVPCVCTYLGSPGVRDNINMDKVFYVTKQCGIAIPGNCGGEQASLDWPH

>MtN5 (XP_003617674.1)
MAHSQGKALAQWMIGALLFAMLAGSLAVQICNIDPNDLKQSCSKFVTGRNPPRADEACCGVLR
RANLPCLCGYKSALTYYGINAKKALALPGQCGLQTPSNC

>NtLTP1 (XP_016433981.1)
MEMVSKIACFVVLCMVVVAPHAEALTCGQVQSSLAPCVPYLLGRGPLGGCCGGVKRLLGAAR
TPADRKTACNCLKSAANTFKGIDMGNAARLPGTCGVNIPYKISPSTDCSKVQ

>NtLTP2 (Q03461.1)
MEMVGKIACFVVLCMVVVAPHAEALSCGQVQSGLAPCLPYLQGRGPLGSCCGGVKGLLGAAK
SLSDRKTACTCLKSAANAIKGIDMGKAAGLPGACGVNIPYKISPSTDCSKVQ

>NtnsLTP2-like (XP_009631888.1)
MEIAGKIACFVVLCMVVAAPCAEAITCGQVTSNLAPCLAYLRNTGPLGRCCGGVKALVNSARTT
EDRQIACTCLKSAAGAISGINLGKAAGLPSTCGVNIPYKISPSTDCSKVQ

>CaLTPI (AAF23458.1)
MLTDFVRGVDCPHAEAVTCGQIQVGVVNCLPYLQNRGPLGRCCGVIKDLLKLCKTPHERRKSC
KCVKTAANTIKGIDFGKAAGLSGVCGVKIPFEISPSVDCSKVK

>CaLTPII (AAF23459.1)
MDMFGKIACFVLLCMVVVAPSAEALSCSQVTSGLAPCLPYLQGRGPLGGCCSGVKDLLAAAKT
PADRKTACTCMKSTANSIKGIDAGKAASIPATCGVNIPYKISPSTDCTKVE

Supplementary Table 3 – Oligonucleotides used in this study

Primer name	Primer Sequence (5'→3')	Used For
pMDC85 LTP XbaI F	AAA AAA TCT AGA ATG GGT AAG AAC AAT ACC ACA	Cloning into pMDC85
pMDC85 LTP AscI R	AAA AAA GGC GCG CC T CAG GAG GGA TTG CCC ACG CC	Cloning into pMDC85
neLTP pET21a-F BamHI	AAA GGA TCC TAT CCT ATA TGC AAC AC	Cloning into pET21a(+)
neLTP pET21a-R XhoI	AAA CTC GAG TGA CAA ACA GGA GGG AT	Cloning into pET21a(+)
